# Mechanical and Insulation Performance of Rigid Polyurethane Foam Reinforced with Lignin-Containing Nanocellulose Fibrils

**DOI:** 10.3390/polym16152119

**Published:** 2024-07-25

**Authors:** Kabirat O. Bello, Ning Yan

**Affiliations:** 1 Graduate Department of Forestry, University of Toronto, 33 Willcocks Street, Toronto, ON M5S 3B3, Canada; kabirat.bello@mail.utoronto.ca; 2 Department of Chemical Engineering and Applied Chemistry, University of Toronto, 200 College Street, Toronto, ON M5S 3E5, Canada

**Keywords:** rigid polyurethane foam, lignin-containing nanocellulose fibrils (LCNF), mechanical properties, thermal conductivity

## Abstract

Isocyanates are critical components that affect the crosslinking density and structure of polyurethane (PU) foams. However, due to the cost and hazardous nature of the precursor for isocyanate synthesis, there is growing interest in reducing their usage in polyurethane foam production—especially in rigid PU foams (RPUF) where isocyanate is used in excess of the stoichiometric ratio. In this study, lignin-containing nanocellulose fibrils (LCNF) were explored as mechanical reinforcements for RPUF with the goal of maintaining the mechanical performance of the foam while using less isocyanate. Different amounts of LCNF (0–0.2 wt.%) were added to the RPUF made using isocyanate indices of 1.1, 1.05, 1.0, and 0.95. Results showed that LCNF served as a nucleating agent, significantly reducing cell size and thermal conductivity. LCNF addition increased the crosslinking density of RPUF, leading to enhanced compressive properties at an optimal loading of 0.1 wt.% compared to unreinforced foams at the same isocyanate index. Furthermore, at the optimal loading, LCNF-reinforced foams made at lower isocyanate indices showed comparable stiffness and strength to unreinforced foams made at higher isocyanate indices. These results highlight the reinforcing potential of LCNF in rigid polyurethane foams to improve insulation and mechanical performance with lower isocyanate usage.

## 1. Introduction

Polyurethanes (PU) are a diverse group of polymers with a wide range of applications as adhesives, coatings, elastomers, and foams [1]. Specifically, rigid polyurethane foams (RPUF) have been extensively used in applications requiring a combination of thermal insulation and structural support [2,3]. This unique combination of properties is due to the closed-cell structure and highly crosslinked polymer network resulting from the reaction of polyols with excess diisocyanate in the presence of catalysts, surfactants, blowing agents, and other additives [4]. However, the production of RPUF is currently heavily reliant on petroleum-based diisocyanates, a component known for its toxicity and environmental unfriendliness as it is synthesized from phosgene as a precursor [5]. The isocyanate (NCO) index defines the ratio of isocyanate to hydroxyl groups present in a formulation. This value, which is typically between 1.05 and 1.2 in RPUF (implying an excess of 5–20% isocyanate groups), significantly influences the chemical structure and properties of the resulting foam through the formation of additional crosslinks and hard segments, which contribute to enhanced foam strength and stiffness [6]. Despite growing environmental and economic concerns, recent efforts to explore non-isocyanate routes still face challenges, such as complex synthesis routes, low mechanical performance, and high processing costs, which continue to limit their large-scale applications [7]. Hence, it is imperative to develop alternative techniques to reduce isocyanate content in foam formulations without compromising essential foam properties. One promising avenue to achieve this is through the incorporation of nanofillers.

Renewable biomass-based fillers have gained substantial attention for reinforcing polyurethane foams owing to their natural abundance, remarkable mechanical properties, and reactive functional groups [8,9,10,11,12,13]. Cellulose nanofiber (CNF), specifically, has been employed to tune the morphology and microstructure of foams, resulting in improved mechanical and thermal properties [10,11,14,15,16,17,18]. CNF with abundant hydroxyl groups is well suited for PU systems since the hydroxyl (OH) groups in CNF can react with isocyanate to form urethane linkages. However, the effectiveness of this modification largely depends on factors like loading amount, polymer-filler compatibility, dispersion quality, and incorporation method. Similar enhancements have been reported for cellulose nanocrystals [19,20], bacterial cellulose [21,22], and micro-sized lignin fillers [23] in polyurethane foams. Faruk et al. [10] explored the reinforcing ability of CNF and lignin fillers in polyurethane foams. Results showed that reinforced foams produced using lower isocyanate content exhibited a similar compressive strength and modulus compared to unfilled foams produced at a higher isocyanate content. However, this study did not provide the isocyanate index used for foam formulation or consider the impact of density on the foam properties, making it challenging to fully understand the extent of filler improvements. Furthermore, production of CNF from biomass feedstock involves an initial chemical bleaching step to remove lignin prior to further mechanical processing [24], which increases the energy/chemical cost and environmental impact of the overall process.

Recent research has reported a new type of nanomaterial, lignin-containing nanocellulose fibrils (LCNF), with additional beneficial properties, such as improved polymer compatibility, hydrophobicity, antioxidation, thermal stability, and ultraviolet absorption due to the residual lignin component compared to pure CNF without lignin [25,26]. LCNF have been effectively utilized to enhance the performance of polymer composites for energy storage devices [27], composites [28,29,30,31,32], and packaging materials [33]. In these applications, lignin acted as a natural coupling agent to form strong interfaces between the hydrophilic nanofibrils and the hydrophobic polymer matrix. Specifically, LCNF have been shown to provide significant improvement to the mechanical performance of pMDI wood adhesive due to the reaction between LCNF-OH groups and pMDI-NCO groups to form polyurethane linkages [34]. Under similar production conditions, LCNF-containing adhesives showed improved adhesive performance and reactivity compared to CNF-containing adhesives, illustrating the functional role of residual lignin in LCNF [35]. This unique nature makes LCNF an interesting filler choice for RPUF synthesis. It is anticipated that the presence of residual lignin in LCNF would offer advantages that could effectively improve the performance of LCNF-reinforced foams compared to their lignin-free counterparts. Additionally, LCNF is more environmentally friendly compared to CNF because it provides energy/chemical savings by increasing production yield from the starting biomass materials and using fewer chemicals.

This study investigated, for the first time, the use of LCNF as a reinforcing filler for RPUF synthesis. Conventional isocyanates and polyols were used due to their prevalent use in the foam industry. We studied the effect of low loadings of LCNF (0–0.2 wt.%) on the physical, mechanical, and thermal properties of RPUF at different NCO indices (1.1, 1.05, 1.0, and 0.95), ranging from above to below the stoichiometric ratio, to evaluate the ability of LCNF to compensate for reduced hard segments at low NCO indices. This study demonstrated an efficient method to reduce isocyanate usage in RPUF while maintaining the mechanical performance of the foam, thus enhancing sustainability and reducing cost.

## 2. Materials and Methods

### 2.1. Materials

Polymeric diphenylmethane diisocyanate (pMDI, Rubinate M) with a 31% NCO content by weight and functionality of 2.7, N, N-dimethylcyclohexylamine (DMCHA), and pentamethyldiethylenetriamine (PMDETA) catalysts were supplied by Huntsman (Arlington, TX, USA). A polyether polyol, polyethylene glycol (PEG 400), with a molecular weight of 400 Da, hydroxyl number of 280.5 mgKOH/g, and functionality of 2, was purchased from Fisher Scientific (Mississauga, ON, Canada). Glycerol with a molecular weight of 92.09 Da, a hydroxyl number of 1827.5 mgKOH/g, and functionality of 3 was used as the crosslinker and purchased from Caledon Laboratories (Georgetown, ON, Canada). Sodium hydroxide and cyclopentane were purchased from Sigma Aldrich (Oakville, ON, Canada). The surfactant, TEGOSTAB B 8460, was supplied by Evonik Corporation (Allentown, PA, USA). Red Cedar bark was supplied by Terminal Forest Products (Richmond, BC, Canada). All chemicals were used as received without further purification.

### 2.2. Lignin-Containing Nanocellulose Fibrils (LCNF) Preparation

LCNF were prepared from Red Cedar bark according to methods previously described in [36]. Briefly, shredded bark fibers were pretreated in 1% NaOH solution with a liquor-to-bark ratio of 10:1 at 90 °C for 2 h to remove extractives. The remaining bark materials were washed repeatedly with an excess amount of water until a clear solution was observed. The treated bark was left to soak in water for 48 h and afterwards was transferred into a supermass colloider (MKZA10-15 J, Masuko Sangyo Co., Ltd., Kawaguchi, Saitama, Japan) for fibrillation at 1500 rpm for 20 passes to obtain an LCNF suspension. Finally, the concentration of LCNF suspension was adjusted to 1 wt.% before making foam.

### 2.3. Polyol Preparation

The base polyol, referred to as Pcontrol, was prepared by using PEG 400 and glycerol at an 80:20 *w*/*w* ratio [37]. Glycerol was used to improve crosslinking density [23,38]. A weighted amount of LCNF suspension was added to Pcontrol to achieve a concentration of 0.1 wt.% and 0.2 wt.% of dry LCNF in the polyol, denoted as LP1 and LP2, respectively. Each polyol dispersion was vigorously mixed with an overhead stirrer at 2000 rpm for 30 min, followed by the removal of water under vacuum using a rotary evaporator.

### 2.4. Preparation of RPUF

RPUF were produced using Pcontrol, LP1, and LP2 polyols at four NCO indices of 1.1, 1.05, 1.0, and 0.95. Relative to the polyol weight, blowing agents (1.5% water and 5% cyclopentane), catalyst (0.08% PMDETA, 0.42% DMCHA), and 2% surfactant were mixed with the respective polyols using an overhead mixer at 2000 rpm for 5 min. Afterwards, the mixture was sonicated for 15 min before mixing at 2000 rpm for 10 s with the calculated weight of pMDI to achieve the required NCO index. The mixture was quickly poured into an open aluminum mould and left to allow the foam to rise freely. Samples were cured at room temperature for 24 h before further characterization was performed. Cured foams were cut into test specimens using a band saw after removing about 1 cm of material from the top and bottom layers. It is important to note that the quantity of blowing agent, catalyst, and surfactant was kept constant between sample batches. Foams produced using Pcontrol, LP1, and LP2 polyols were denoted as PUF, PUF1, and PUF2, respectively. Within each sample set, foams produced at different NCO indices are referred to by the nomenclature “foam name/NCO index”; for example, PUF/1.1 refers to control foam produced at an NCO index of 1.1.

### 2.5. Characterization

#### 2.5.1. Polyol Characterization

The hydroxyl (OH) value of Pcontrol, LP1, and LP2 polyols was determined using the standard esterification method using phthalic anhydride according to ASTM D4274 [39]. The viscosity of the polyols was also measured with a digital viscometer (Model DV-E, Brookfield Engineering Laboratories, Middleboro, MA, USA) at room temperature (25 °C) using spindle no. 62. The OH value and viscosity reported were calculated as the average of three replicates.

#### 2.5.2. Physical Properties of RPUF

The apparent density of the foams was determined according to ASTM D1622 [40]. Open cell content was measured using a pycnometer (Model AccuPyc II 1340, Micromeritics Instrument Corporation, Norcross, GA, USA) according to ASTM D6226 [41]. For both tests, the average of three test values was reported.

#### 2.5.3. Gel Content of RPUF

The crosslinking density of the foam samples was determined using the gel content test according to previously reported methods [42,43,44]. Foam samples were refluxed in dimethyl formamide (DMF) for 24 h. Upon removal, samples were dried in the oven at 60 °C until a constant weight was achieved. The gel content (%) was calculated as the ratio of the mass of the dried sample to that of the initial sample multiplied by 100.

#### 2.5.4. Fourier Transform Infrared Spectroscopy

The chemical structure of RPUF was determined using a Fourier Transform Infrared (FTIR) Spectrometer (Spectrum Two, Perkin Elmer, Shelton, CT, USA). FTIR spectra in transmission mode were recorded at room temperature from 4000 to 400 cm^−1^ with 32 scans at a resolution of 4 cm^−1^.

#### 2.5.5. Scanning Electron Microscopy

The morphology and cell size distributions of RPUF were examined using a Scanning Electron Microscope (SEM) (JSM-6610LV, JEOL Ltd., Akishima, Tokyo, Japan) at an accelerating voltage of 15 kV. Foam samples were placed on SEM mounts using carbon tape, and the surface was sputter-coated with gold. The mean cell sizes were calculated from an average of 100 cells captured in the SEM images.

#### 2.5.6. Laser Confocal Microscopy

Confocal Microscopy was used as an additional characterization technique to evaluate the distribution of LCNF in the foam matrix. This technique utilizes laser excitation and typically involves staining samples with dyes before analysis [45]. However, due to the natural autofluorescence of lignin resulting from endogenous fluorophores, specifically monolignols, foam samples PUF1 and PUF2 did not require staining prior to analysis [45,46].

Fluorescence images were obtained using a Laser Confocal Microscope (Stellaris 5, Leica Microsystems, Wetzlar, Germany) with an excitation wavelength of 561 nm under a 10× objective. All foam samples were scanned parallel to the free-rise direction, and images were analyzed using ImageJ software (1.53t).

#### 2.5.7. Mechanical Tests

The compressive strength and modulus of foam samples measuring 51 × 51 × 25.4 mm^3^ were determined according to ASTM D1621 [47] using a universal testing machine (Model 3367, Instron, Norwood, MA, USA) with a 30 kN load cell and a crosshead speed of 2.5 mm/min. Samples were placed between two plates, and the compressive strength at 10% sample deformation strain was recorded. The average result of three tests was reported.

#### 2.5.8. Thermal Tests

The thermal conductivity value (λ) of foam samples measuring 203.2 mm × 203.2 mm × 27 mm was determined using a heat flow meter (FOX200, TA Instruments, New Castle, DE, USA) equipped with a cooling unit according to ASTM C518-10 [48]. The samples were tested between two isothermal plates maintained at 20 °C (upper plate) and 45 °C (bottom plate).

The thermal degradation behavior of foam samples was analyzed using a Thermogravimetric Analyzer (TGA) (Q500, TA Instruments, New Castle, DE, USA). Samples were heated from room temperature to 700 °C at a rate of 10 °C min^−1^ under a nitrogen atmosphere.

#### 2.5.9. Statistical Analysis

The normality of the data distribution was analyzed using the Shapiro-Wilk test due to its effectiveness for small sample sizes. The test confirmed that the distribution followed a normal distribution at a 5% level of significance. The statistical variation among samples for the mechanical properties, thermal conductivity, and density was analyzed using the analysis of variance (ANOVA)-Tukey test at a confidence level of 95% (*p* < 0.05). OriginPro software (2023) was used for all statistical analyses.

## 3. Results and Discussion

### 3.1. Polyols

Optical microscope images of Pcontrol, LP1, and LP2 are shown in Figure S1a–c, respectively. It was observed that LCNF were highly dispersed, with small amounts of micro-sized fiber clusters visible in the polyol mixture. Achieving homogeneous filler dispersions in polyol mixtures is important for improving cell structure and, consequently, the thermal and mechanical performance of RPUF [19,23]. The homogenous LCNF/polyol dispersion achieved in this study was attributed to a combination of factors, such as the choice of polyether polyol (low molecular weight polyethylene glycol), which has been previously shown to provide better lignin dispersion compared to polypropylene oxide-based polyols due to a lower viscosity and a higher number of hydrogen donor sites [49]; low concentration of nanofillers [11]; and a combination of sonication and high-shear mixing procedures [19,23].

The viscosity of Pcontrol, LP1, and LP2 polyols is presented in Table S1. It can be observed that the viscosity of the LCNF-containing polyols increased with the level of LCNF addition and was higher than that of the control polyol, likely due to hydrogen bonding interactions between LCNF and the polyol mixture [20,50]. However, the viscosity of both LP1 and LP2 was still well within the acceptable range of 200–50,000 MPa·s for rigid polyurethane foams [51]. The FTIR spectra (Figure S2a) showed no apparent changes in the functional groups due to the very low level of LCNF addition. Also, the hydroxyl value (OHv) (Table S1) did not significantly change due to LCNF addition since the hydroxyl values in LCNF could not be measured using the standard esterification method. These results were consistent with other studies that had incorporated low loadings of nanocellulose [19,20] and kraft lignin [23] in polyol.

### 3.2. Influence of LCNF on RPUF Properties

#### 3.2.1. Chemical Structure of RPUF

The FTIR spectra of the control foam, PUF, and LCNF-reinforced foams, PUF1 and PUF2, at all NCO indices showed similar characteristic peaks of polyurethane foams, as seen in Figure S2b. Urethane linkages could be readily observed due to the N-H stretching and bending vibration absorptions around 3300 cm^−1^ and 1520 cm^−1^, C=O stretching vibration around 1720 cm^−1^, and C-N stretching around 1200 cm^−1^ [17]. The presence of a weak N=C=O band centered around 2270 cm^−1^ indicates the presence of residual isocyanate in the foams. The intensity of this peak, however, reduced as the isocyanate index reduced. In addition, no new functional groups were observed in PUF1 and PUF2 foams. This was attributed to the low amounts of LCNF present in the foam [19,20].

#### 3.2.2. Density of RPUF

**Figure 1:**
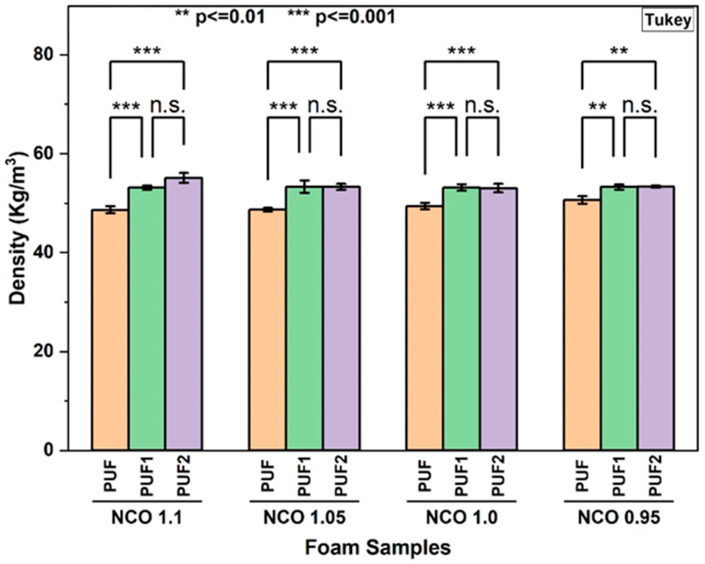
Density of foams at different NCO indices (n.s.—not significant).

Density is a major parameter that influences the mechanical properties of RPUF. Figure 1 illustrates the effect of varying NCO indices and LCNF content on the density of the foams. The average density of all RPUF ranged between 49 and 55 kg/m^3^, which is within the suitable range of 30–100 kg/m^3^ for application as lightweight thermal insulation in appliances and buildings [1]. A slight increase in the density of LCNF-reinforced foams was observed when compared to the density of the control foams at the same isocyanate index. Specifically, the incorporation of 0.1 wt.% and 0.2 wt.% of LCNF led to an average density increase ranging between 5–10% and 7–11% for PUF1 and PUF2, respectively, compared to the control foams for all NCO indices. This implied that LCNF affected cell nucleation and growth, leading to a denser foam structure [23]. This was further supported by SEM images showing higher heterogeneity and reduced cell size as the content of LCNF increased.

However, within each foam formulation (PUF, PUF1, or PUF2), no significant variation in density was observed as the NCO index decreased from 1.1 to 0.95 (Figure S3). This was likely because the variation in the isocyanate indices was not substantial enough to trigger significant changes in the foam density [13]. This indicated that the reduction in the isocyanate index did not significantly impact the overall cell nucleation and growth enough to cause changes in foam density. Several of the literature studies also demonstrated that the density of RPUF was not always proportional to the isocyanate index or filler content and depended on the specific formulation [52,53]. One study [13] showed that the incorporation of 0.5 wt.% cellulose nanocrystals in the foam formulation did not significantly affect foam density but instead resulted in increased foam heterogeneity. Other studies reported either a decrease in foam density as the cellulose nanofibril content in the foam increased from 0.1 wt.% to 0.4 wt.% [11], or insignificant changes to the foam density with the addition of up to 3 wt.% of nanocellulose [54]. Whereas here, the incorporation of LCNF resulted in a slight increase in the foam density. In addition to the formation of smaller, densely packed cells, this could also be attributed to the high reactivity of LCNF with isocyanate in the polymer matrix to form a more compact and rigid foam structure, contributing to an increase in density [23,38].

#### 3.2.3. Cell Morphology of RPUF

**Figure 2:**
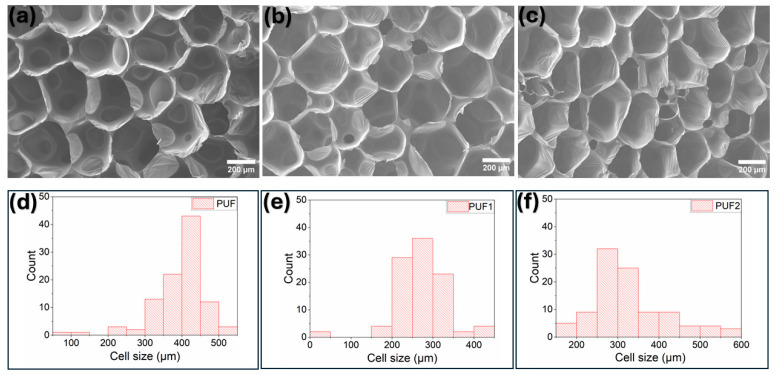
SEM images and cell size distribution: (**a**,**d**) PUF/1.1; (**b**,**e**) PUF1/1.1; and (**c**,**f**) PUF2/1.1.

The cell morphology of RPUF has direct implications on their mechanical and thermal performance [3]. The microstructural analysis of the control foam, PUF, and those containing 0.1 wt.% and 0.2 wt.% LCNF, PUF1 and PUF2, showed a predominantly polygonal closed-cell structure [38], as seen in Figure 2a–c. The mean cell sizes are given in Table 1. The addition of LCNF significantly impacted the cell morphology, producing a more heterogeneous microstructure (Figure 2d–f). At an NCO index of 1.1, the mean cell size reduced from 397.6 µm for control PUF to 274.1 µm and 332.1 µm for PUF1 and PUF2 samples, respectively. Although PUF2/1.1 exhibited a higher cell size compared to PUF1/1.1, it was still significantly lower than the control. This trend was also observed in foams produced at NCO indices of 1.05, 1.0, and 0.95. The decrease in the cell size of LCNF-reinforced foams confirmed the nucleating effect of LCNF, possibly resulting from the reduction in the coalescence of bubbles during the foaming process [9]. This implied that the optimum loading level for the foam formulation used in this study was around 0.1 wt.%. At the optimum level, the well-dispersed nanofillers significantly lowered nucleation-free energy, consequently leading to a finer cell structure and reduced thermal conductivity [55].

Within each sample group (PUF, PUF1, or PUF2), a reduction in the NCO index from 1.1 to 0.95 did not considerably affect the average cell size (Table 1). This suggested that the reduction in the isocyanate index did not significantly impact cell nucleation and growth [56], which was consistent with the trend observed in the foam density. Similar observations were reported in a prior report [6] on cellulose fiber-reinforced rapeseed oil-based polyurethane foams produced at varying isocyanate indices. Hence, representative SEM images and cell size distribution of the foams made at an NCO index of 1.1 are shown in Figure 2. Furthermore, there was no significant difference in the closed-cell content of PUF1 samples from that of the control foams (Table 1), confirming a relatively well-dispersed distribution of LCNF within the cell wall and cell struts. However, at a higher LCNF loading level, PUF2 samples showed a slight decrease in the closed-cell content compared to the control foams. This could be due to LCNF disrupting the foaming process, resulting in a less homogenous and distorted cell structure, as shown in the SEM images (Figure 2c). This has also been observed in reinforced polyurethane foams at loading levels above the optimum [23,38].

#### 3.2.4. Confocal Microscope Characterization of RPUF

**Figure 3:**
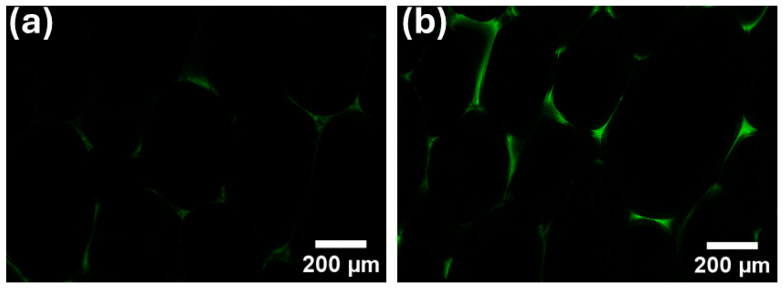
Confocal images of LCNF-reinforced foams (**a**) PUF1 and (**b**) PUF2 at NCO index of 1.1.

Since lignin can emit fluorescence upon light excitation, LCNF can be used as a fluorescent marker for imaging of foams. The structure of the control samples (PUF) without fluorescent markers (LCNF) could not be visualized under the microscope. Figure 3a,b shows confocal images of LCNF-reinforced foams, PUF1 and PUF2, respectively.

Due to the limitation in the fluorescence detection resolution, it was not possible to visualize individual LCNF in the foam matrix. However, the fluorescence emitted by LCNF was detected in PUF1 and PUF2 in the form of a light green color, which intensified as the LCNF content increased from 0.1 wt.% to 0.2 wt.% to enable imaging of the cell structure and visualization of LCNF distributions in the foam matrix. It was observed that LCNF was mainly distributed in the cell walls and struts. The images were not significantly different for foams produced at lower NCO indices; hence, representative images of foams with an NCO index of 1.1 were reported.

#### 3.2.5. Gel Content of RPUF

**Figure 4:**
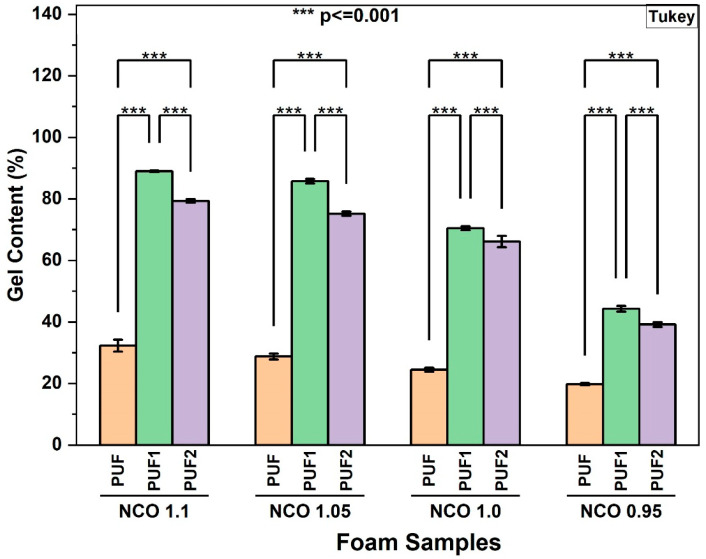
Gel content of foams at different NCO indices.

The gel content experiment was used to confirm the formation of stable crosslinked networks in the RPUF samples. As shown in Figure 4, the gel content of control samples, PUF, was significantly lower than that of LCNF-reinforced samples, PUF1 and PUF2, for all NCO indices. The increase in gel content showed that LCNF reacted with isocyanate to form higher crosslinking networks in foams. Furthermore, it was observed that an increase in the LCNF concentration from 0.1 wt.% to 0.2 wt.% led to a decrease in the gel content of the samples. This further confirmed the optimum LCNF loading for the formulation used in this study to be around 0.1 wt.%, above which the presence of LCNF began to interfere with the foaming mechanism, leading to the formation of less stable crosslinked networks.

Within each sample group (PUF, PUF1, and PUF2), it was observed that the gel content decreased as the NCO index decreased from 1.1 to 0.95 (Figure S4). This confirmed that there was a strong correlation between the isocyanate content and the crosslinking density of the polyurethane foams, which was expected since there was less isocyanate available to form crosslinks when the NCO index was reduced [44].

#### 3.2.6. Compressive Strength and Modulus of RPUF

**Figure 5:**
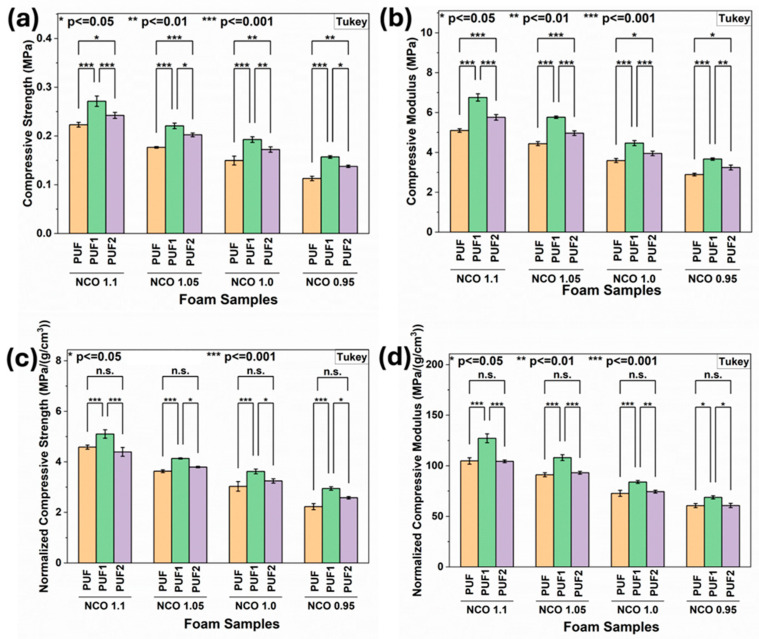
(**a**) Compressive strength; (**b**) Compressive modulus; (**c**) Normalized Compressive strength; and (**d**) Normalized Compressive modulus of foams at varying NCO indices (n.s.—not significant).

The compressive strength and modulus results of PUF, PUF1, and PUF2 samples grouped by sample type are given in Figure S5. As expected, when the NCO index reduced from 1.1 to 0.95, RPUF samples showed decreased compressive strength and modulus for all sample groups. This result was expected because the amount of isocyanate determines the extent of crosslinking and the fraction of hard segments (urethane and urea linkages) in the polyurethane network, ultimately affecting the strength and stiffness of the resulting foam [6]. The incorporation of LCNF in the foam matrix resulted in significant improvements in the compressive strength and modulus of PUF1 and PUF2 samples compared to control PUF samples at all NCO indices, as shown in Figure 5a,b. This could be attributed to the higher crosslinking density in LCNF-reinforced foams, as observed in the gel content results in Figure 4. Furthermore, it was observed that increasing the LCNF concentration from 0.1 wt.% to 0.2 wt.% reduced the compressive properties of the samples significantly. The decrease in compressive properties of PUF2 samples compared to PUF1 samples was attributed to the formation of less stable crosslinking networks due to LCNF interfering with the crosslinking reactions between the polymer chains at higher loadings. Reduction in the mechanical performance of rigid foams at nanofiller loadings above the optimum content has been generally seen in previous studies of pure nanocellulose fillers [11,19].

The compressive properties of polyurethane foam are directly related to its density [1]. Hence, the compressive strength and modulus were normalized against the foam density to ensure that comparisons are based on the material’s inherent properties rather than variations in density. At all NCO indices, PUF1 samples showed significant improvement in normalized compressive strength and modulus compared with control foam (PUF), as shown in Figure 5c,d. However, no significant improvement in normalized compressive strength and modulus was observed when PUF2 samples were compared with control PUF samples at all NCO indices. This further confirmed that the loading level of 0.1 wt.% was the optimum content for the LCNF in these formulations. The improved normalized compressive strength and modulus observed in PUF1 samples indicated that low LCNF loadings effectively reinforced the foam matrix beyond the densification effect.

**Figure 6:**
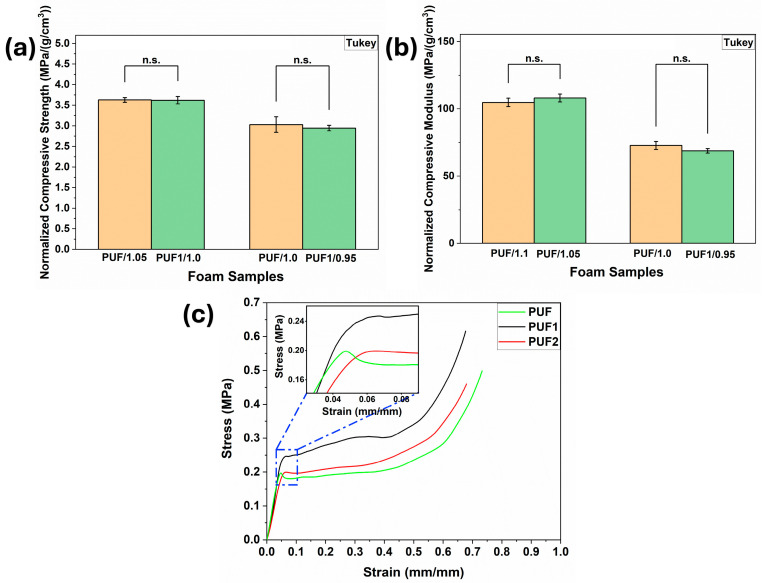
Effect of LCNF incorporation on (**a**) Normalized compressive strength; (**b**) Normalized compressive modulus of RPUF (n.s.—not significant); and (**c**) Representative compressive stress and strain curve of foam samples at an NCO index of 1.1.

Furthermore, it was observed that PUF1/1.0 and PUF1/0.95 samples produced with a lower isocyanate content (about 4.5% reduction) showed comparable normalized compressive strength to control foams, PUF/1.05 and PUF/1.0, with a higher isocyanate content, respectively (Figure 6a). Similarly, the normalized compressive modulus of PUF1/1.05 and PUF1/0.95 samples was not significantly different from that of control foams with a higher NCO index, PUF/1.1 and PUF/1.0, respectively, as shown in Figure 6b. This result indicated the reinforcing effect of LCNF in compensating for the reduction in hard segments due to a lesser amount of isocyanate precursor in the formulation.

As demonstrated in Figure 6c, all foams in this study exhibited linear-elastic deformation at a low strain level (<10%), followed by a plateau region likely resulting from the buckling and fracture of the cell wall struts. At a higher strain level (>45%), foam densification resulted in a steady increase in stress. This phenomenon has been widely reported by other studies on polyurethane foams [57,58,59]. Furthermore, the yield strength of control PUF occurred at a lower strain level and was accompanied by lower compressive strength compared with LCNF-reinforced foams PUF1 and PUF2, as shown in the Figure 6c inset. This result further confirmed the reinforcing effect of LCNF.

#### 3.2.7. Thermal Conductivity of RPUF

**Figure 7:**
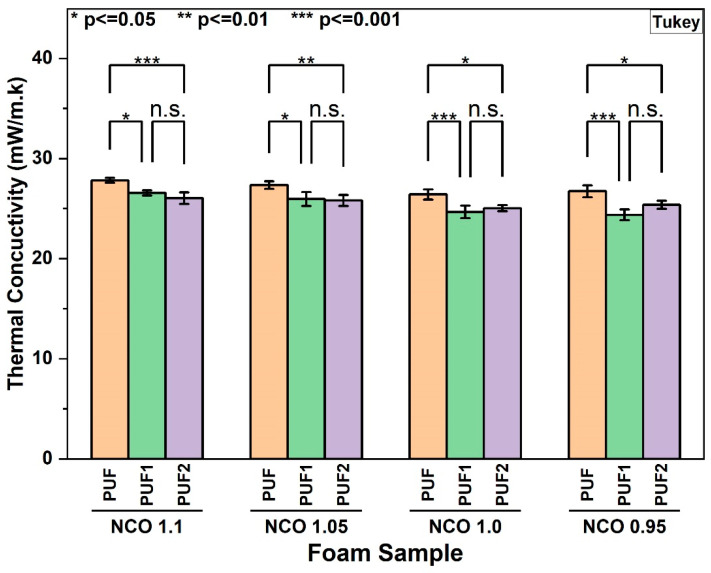
Thermal Conductivity of foam samples at different NCO indices (n.s.—not significant).

The thermal conductivity of RPUF is primarily influenced by the cell size, cell structure (open/closed cells), and the choice of blowing agent [3,19]. Figure 7 shows that the thermal conductivity of control foam PUF and LCNF-containing foams, PUF1 and PUF2, produced at different NCO indices ranged between 24.4 and 27.8 mW/m K. These values fall within the acceptable range for thermal insulation applications [38,60]. The incorporation of LCNF significantly reduced the thermal conductivity of the foams within the range of 4.5% to 10% for all NCO index levels, as shown in Figure 7. Given that the amount of blowing agents and surfactant was kept constant in the formulation, the improvement in the thermal insulation properties of LCNF-reinforced foams could be attributed to the significant reduction in the average cell size due to the nucleating effect of LCNF and the high closed-cell content of the foams (Table 1). PUF2 samples, however, showed no significant difference in thermal conductivity values compared with PUF1 samples at all NCO indices. This result was likely because other factors, such as density and blowing agent, outweighed the influence of cell size and closed-cell content when the LCNF content was increased from 0.1 wt.% to 0.2 wt.% in the foam. Although some studies [19,38] have attempted to achieve a reduction in the thermal conductivity of RPUF by using higher nanofiller loadings, it has been at the expense of mechanical properties. Here, our findings indicated the potential of a very low concentration of LCNF reinforcement in enhancing the thermal insulation properties of RPUF without detrimental effects on the foam compressive properties.

#### 3.2.8. Thermal Stability of RPUF

**Figure 8:**
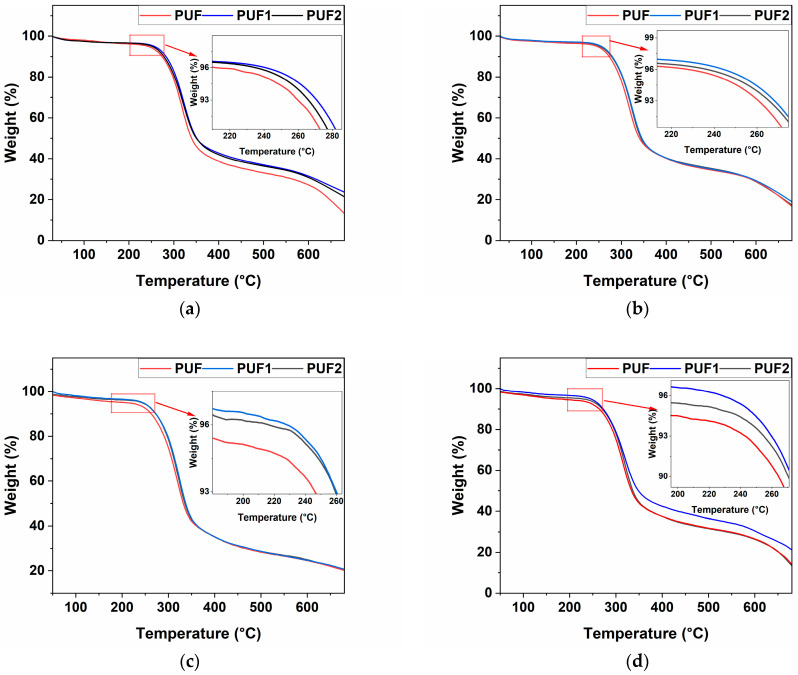
TGA curves of RPUF at NCO index of (**a**) 1.1; (**b**) 1.05; (**c**) 1.0; and (**d**) 0.95.

The TGA and derivative TGA (DTG) curves of the foam samples made at all NCO indices are presented in Figure 8 and Figure S6, respectively. Table 2 shows the decomposition temperature at 5% weight loss (T5%), the temperature at maximum weight loss rate (Tm), and the char residue content at 700 °C. The decomposition behavior of all samples was observed in two stages. The first stage involved the decomposition of the soft segment between 200 °C and 300 °C, while the second stage was related to the decomposition of the hard segment above 300 °C [12]. As shown in Figure 8 and Table 2, LCNF-reinforced samples, PUF1 and PUF2, showed slightly higher T5%, Tm, and char residue content compared with control PUF samples at all NCO indices. This observed increase in thermal stability of PUF1 and PUF2 samples further confirmed the presence of chemical interactions between LCNF and isocyanate, which resulted in a more crosslinked polymer network. This was previously shown in the gel content results in Figure 4. Higher crosslinking density in RPUF generally enhances thermal stability because the more densely interconnected polymer network requires more energy to break down [8,12].

A slight reduction in T5%, Tm, and char residue content was observed in PUF2 samples compared to PUF1 samples at all NCO indices. This was likely because the LCNF content was above the optimum, which interfered with the chemical crosslinking reactions, leading to less stable networks. This trend was observed throughout this study.

With respect to the NCO index, the thermal properties T5% and Tm of the RPUF decreased as the NCO index reduced from 1.1 to 0.95. This result was expected because the presence of less isocyanate in a foaming reaction reduced the crosslinking density and, ultimately, the thermal stability of foams [42].

## 4. Conclusions

The impact of incorporating a low amount of renewable LCNF fillers (0.1 wt.%–0.2 wt.%) in rigid polyurethane foam performance was evaluated at different NCO indices. LCNF-reinforced foams had lower cell sizes and induced slight increases in foam density compared with control foams without LCNF. Reduced cell sizes and high closed-cell contents were beneficial in lowering the thermal conductivity of the reinforced foams. The introduction of LCNF also afforded significant improvements in normalized compressive modulus and strength, crosslinking density, and thermal stability of foams at the optimum content of 0.1 wt.% addition level because of more uniform nanofibril dispersion, reinforcement of the cell wall/struts, and interactions between LCNF-OH groups and isocyanate-NCO groups. Additionally, at the optimum addition of 0.1 wt.%, LCNF-reinforced foams produced at a lower isocyanate content (4.5% reduction) exhibited comparable normalized compressive strength and stiffness to control foams produced at a higher isocyanate content, demonstrating their ability to compensate for the hard segment reduction at a lower isocyanate usage. This offers significant environmental and cost benefits by reducing the reliance on isocyanates, which are often derived from non-renewable petroleum sources with hazardous effects on human health. Additionally, using LCNF produced from unbleached, renewable, and abundant tree bark provides energy/chemical cost savings, which enhances the overall sustainability profile of these foam materials.

## Figures and Tables

**Table 1:** Closed Cell Content and Average Cell Size.

Sample	NCO Index	Closed Cell Content (%)	Average Cell Size (µm)
**PUF**	1.1	90.4 ± 0.6	397.6 ± 66
	1.05	89.9 ± 0.9	377.8 ± 41
	1.0	90.1 ± 2.7	385.6 ± 48
	0.95	89.5 ± 1.2	389.6 ± 79
**PUF1**	1.1	90.1 ± 1.6	274.1 ± 61
	1.05	90.4 ± 0.6	265.2 ± 56
	1.0	90.3 ± 2.1	280.5 ± 46
	0.95	89.7 ± 1.2	280.1 ± 58
**PUF2**	1.1	87.6 ± 1.9	332.1 ± 82
	1.05	88.4 ±0.4	322.5 ± 62
	1.0	87.5 ± 0.5	312.9 ± 75
	0.95	86.9 ± 1.3	346.7 ± 90

**Table 2:** Thermal Stability of Foams.

Sample	T5% (°C)	Tm (°C)	Residue (%)
**PUF/1.1**	242.5	318.3	13.2
**PUF1/1.1**	256.8	324.1	22.2
**PUF2/1.1**	251.5	322.4	19.4
**PUF/1.05**	240.5	314.3	14.0
**PUF1/1.05**	255.8	322.4	16.7
**PUF2/1.05**	251.0	322.1	15.1
**PUF/1.0**	218.9	316.1	9.8
**PUF1/1.0**	245.2	322.0	18.9
**PUF2/1.0**	242.1	320.4	9.9
**PUF/0.95**	205.4	311.1	19.4
**PUF1/0.95**	244.3	316.8	20.0
**PUF2/0.95**	224.5	316.6	19.5

## Data Availability

Data is contained within the article or supplementary material.

## Supplementary Materials

The following supporting information can be downloaded at: https://www.mdpi.com/article/10.3390/polym16152119/s1, Figure S1: Optical microscope images of polyol mixture (a) Pcontrol, (b) LP1, and (c) LP2; Table S1: Viscosity of LCNF-polyol; Figure S2: (a) FTIR spectra of polyol—Pcontrol, LP1, and LP2; (b) FTIR spectra of foams at NCO indices of 1.1, 1.05, and 1.0; Figure S3: Density of foams grouped by foam type (n.s.—not significant); Figure S4: Gel content of foams grouped by foam type; Figure S5: (a) Compressive strength, (b) Compressive modulus, (c) Normalized compressive strength, and (d) Normalized compressive modulus of foams grouped by foam type; Figure S6: DTG curves of RPUF at NCO indices of (a) 1.1, (b) 1.05, (c) 1.0, and (d) 0.95.
