# Supplementary material for: Mechanical and Insulation Performance of Rigid Polyurethane Foam Reinforced with Lignin-Containing Nanocellulose Fibrils
Source: Polymers (Basel). 2024 Jul 25;16(15):2119. doi: 10.3390/polym16152119 (PMC11313736; doi:10.3390/polym16152119)
Supplement: Supplementary file 1 [file polymers-16-02119-s001.zip › polymers-3097029-supplementary.pdf]

Supporting information

Mechanical and Insulation Performance of Rigid Polyurethane Foam Reinforced with Lignin-Containing Nanocellulose Fibrils

Kabirat O. Bello [1] and Ning Yan [1,2,*]

[1] Graduate Department of Forestry, University of Toronto, 33 Willcocks Street, Toronto, ON M5S 3B3, Canada
[2] Department of Chemical Engineering and Applied Chemistry, University of Toronto, 200 College Street, Toronto, ON M5S 3E5, Canada
* Correspondence: ning.yan@utoronto.ca

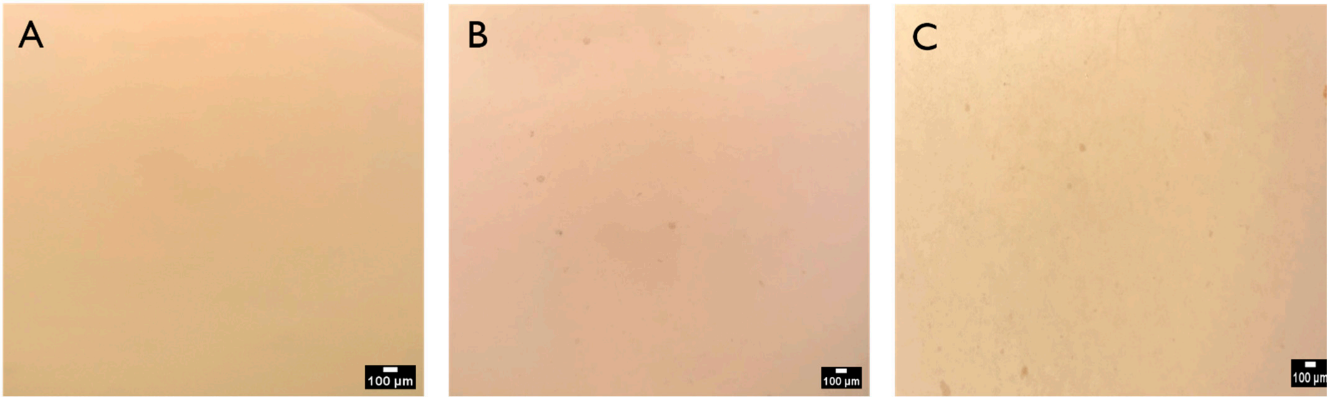

Figure S1. Optical microscope images of polyol mixture a) Pcontrol; b) LP1; and c) LP2

Table S1. Viscosity of LCNF-polyol

Sample	Pcontrol	LP1	LP2
**Viscosity (mPa·s) @ 25 °C**	212.0	272.9	521.0
**Hydroxyl Value (mgKOH/g)**	273.1	270.4	271.2

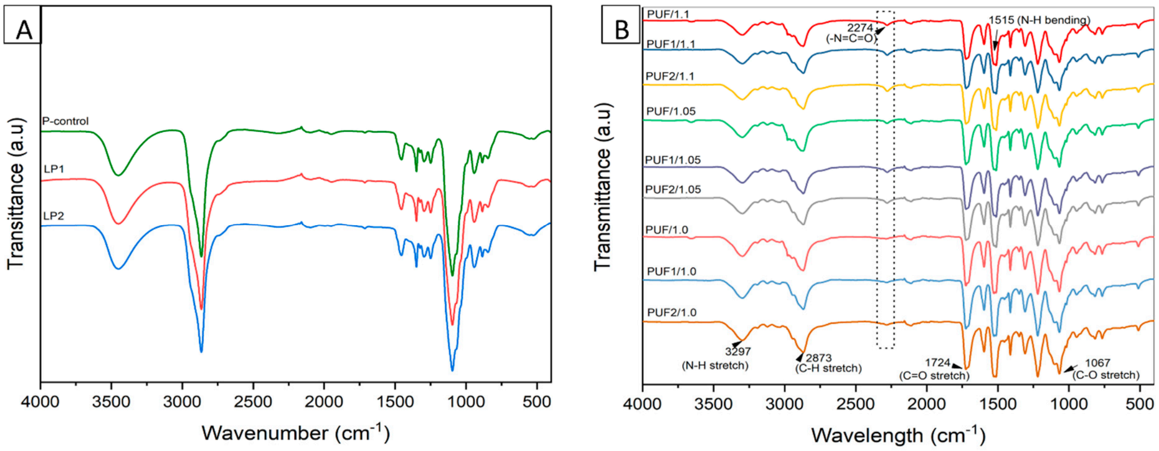

Figure S2. (a) FTIR spectra of polyol - Pcontrol, LP1 and LP2; (b) FTIR spectra of foams at NCO index of 1.1, 1.05 and 1.0

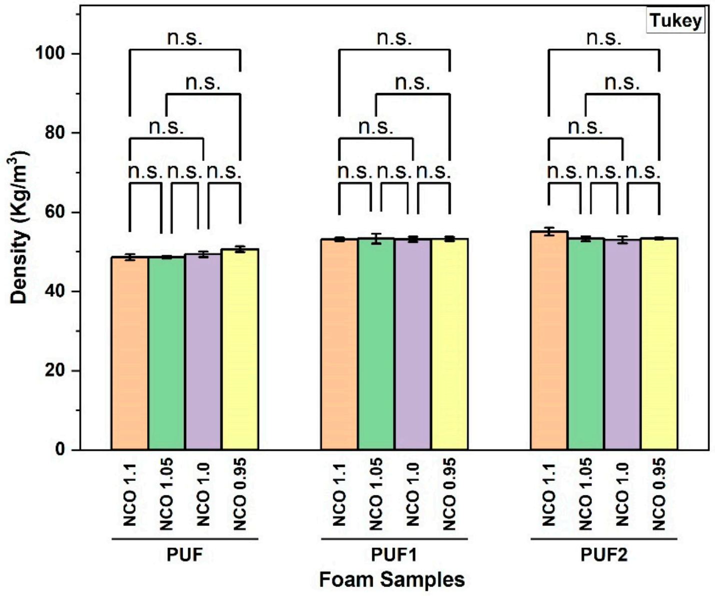

Figure S3. Density of foams grouped by foam type (n.s – not significant)

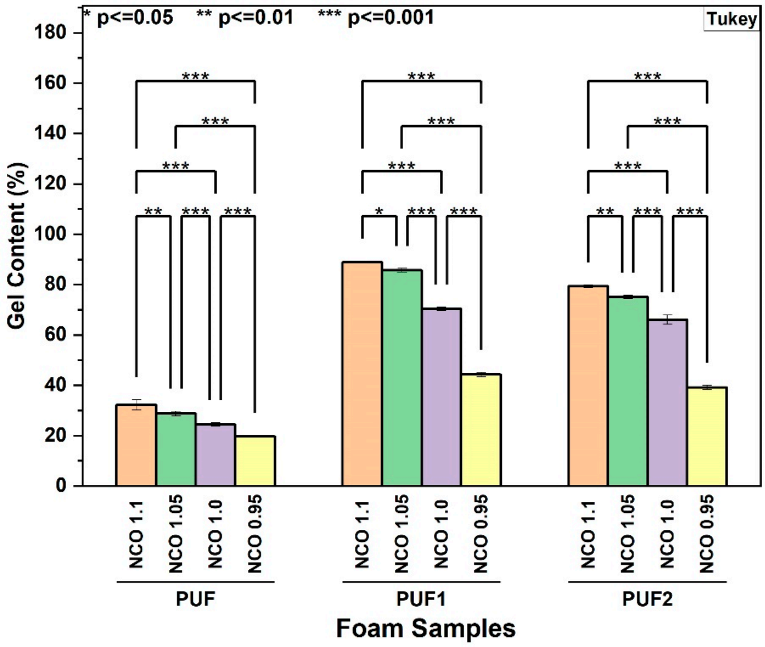

Figure S4. Gel content of foams grouped by foam type

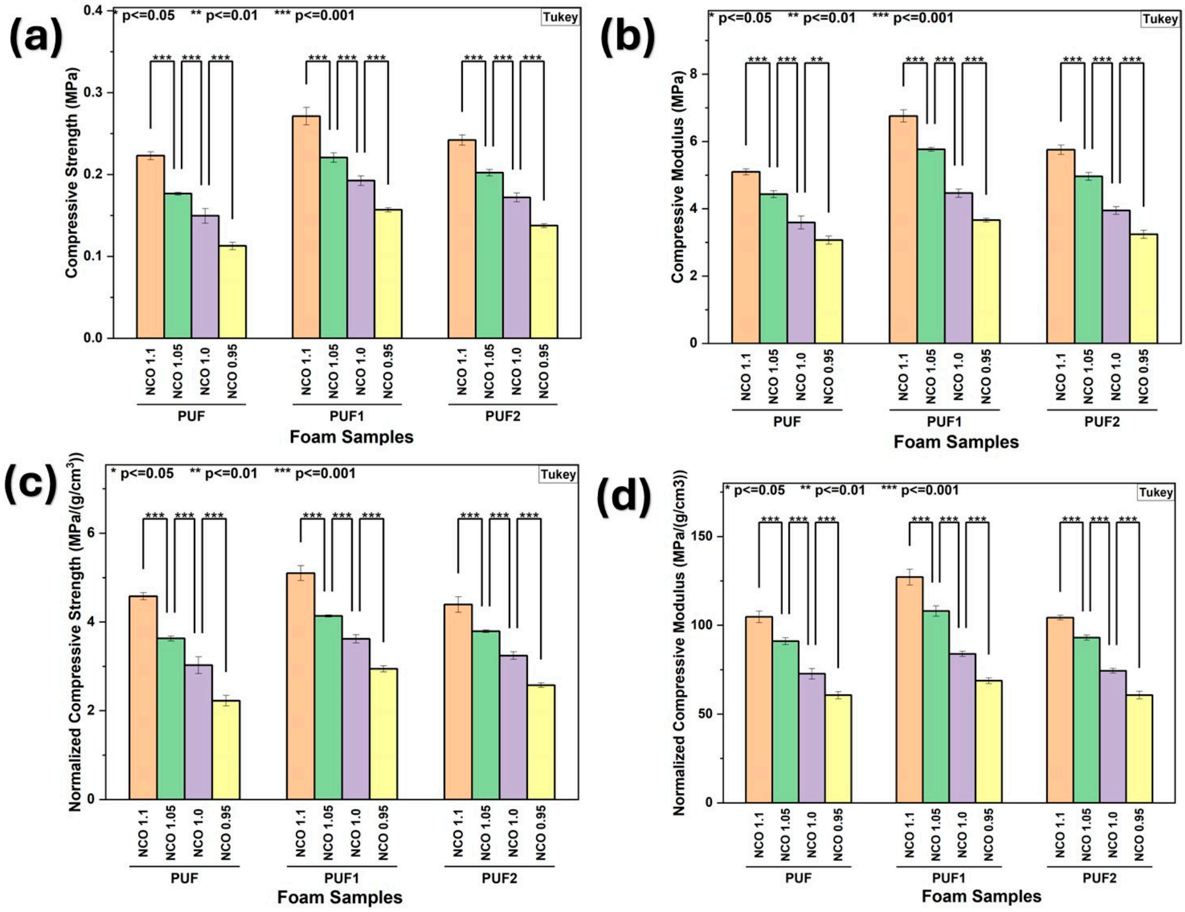

Figure S5. a) Compressive strength; b) Compressive modulus; c) Normalized compressive strength; and d) Normalized compressive modulus of foams grouped by foam type

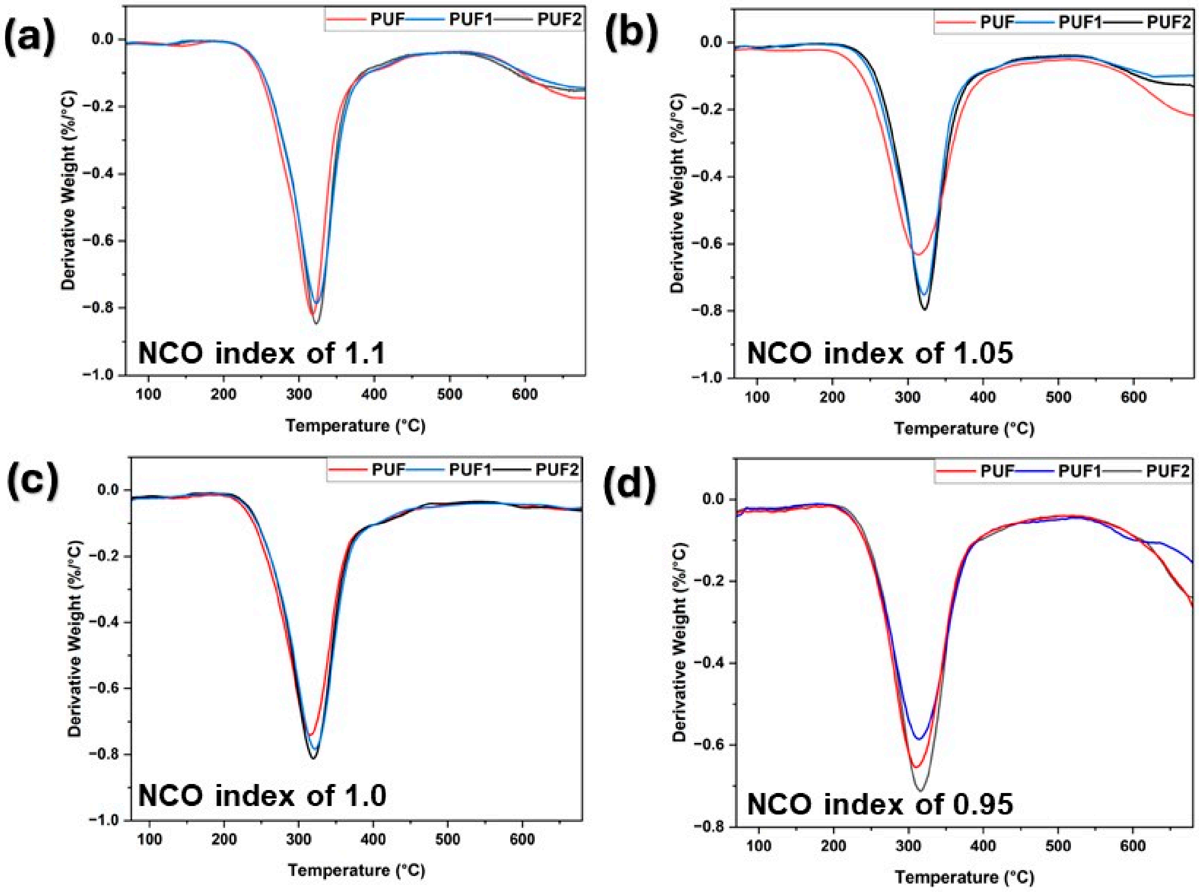

Figure S6. DTG curves of RPUFs at NCO index of a) 1.1; b) 1.05; c) 1.0; and d) 0.95
